# Differential Difficulties in Perception of Tashlhiyt Berber Consonant Quantity Contrasts by Native Tashlhiyt Listeners vs. Berber-Naïve French Listeners

**DOI:** 10.3389/fpsyg.2016.00209

**Published:** 2016-03-01

**Authors:** Pierre A. Hallé, Rachid Ridouane, Catherine T. Best

**Affiliations:** ^1^Laboratoire Phonétique et Phonologie, Centre National de la Recherche ScientifiqueParis, France; ^2^Laboratoire Mémoire et Cognition, Institut National de la Santé et de la Recherche MédicaleParis, France; ^3^Haskins LaboratoriesNew Haven, CT, USA; ^4^MARCS Institute and School of Humanities and Communication Arts, University of Western SydneySydney, NSW, Australia

**Keywords:** nonnative speech perception, Tashlhiyt Berber, French, geminate obstruents, timing perception

## Abstract

In a discrimination experiment on several Tashlhiyt Berber singleton-geminate contrasts, we find that French listeners encounter substantial difficulty compared to native speakers. Native listeners of Tashlhiyt perform near ceiling level on all contrasts. French listeners perform better on final contrasts such as *fit-fitt* than initial contrasts such as *bi-bbi* or *sir-ssir*. That is, French listeners are more sensitive to silent closure duration in word-final voiceless stops than to either voiced murmur or frication duration of fully voiced stops or voiceless fricatives in word-initial position. We propose, tentatively, that native speakers of French, a language in which gemination is usually not considered to be phonemic, have not acquired quantity contrasts but yet exhibit a presumably universal sensitivity to rhythm, whereby listeners are able to perceive and compare the relative temporal distance between beats given by successive salient phonetic events such as a sequence of vowel nuclei.

## Introduction

Cross-linguistic studies of nonnative speech perception—which usually bear on the perception of sublexical units—help us understand the mechanisms that underpin the early stages of pre-lexical speech perception. Pre-lexical processes are the least likely to be biased by such top-down effects as lexical feedback. Some early-stage mechanisms seem to hold universally across languages in their principles but may vary from one language to the other in their specific tunings. Such is the case of categorical perception. And indeed, most of the work accomplished so far in the domain of nonnative speech perception has dealt with the issue of categorization. The models elaborated to account for the observed patterns of nonnative speech perception generally try to formalize how the nonnative phones are categorized or not in terms of native categories (PAM: Best, 1995; L2LP: Escudero, 2005, 2009), and accordingly, how well various nonnative phonemic contrasts may be discriminated, or how difficult it may be to acquire new phonetic categories in the process of learning a second language (SLM: Flege, 1995; PAM-L2: Best and Tyler, 2007; L2LP: Escudero, 2009). Interestingly, the nonnative speech enterprise has not focused equally on the various dimensions of speech sounds. For one thing, the main focus has generally been on consonants, more so than on vowels or tones (see Tyler et al., 2014, for an overview of this asymmetric situation; for tones, see Hallé et al., 2004; So and Best, 2014). Second, most studies have dealt with the perception of single segments rather than segment sequences such as clusters (but see Hallé and Best, 2007; Best and Hallé, 2010).

Yet another dimension may be viewed as somewhat neglected: segmental quantity, that is, vowel or consonant distinctive *duration*. Most of the cross-linguistic studies on the perception of phonemic geminate consonants concern second *language learning*, for example the difficulties encountered by English or Korean learners of Japanese with Japanese geminates in either production or perception (e.g., Hayes, 2002; Hardison and Saigo, 2010; Sadakata and McQueen, 2011; Sonu et al., 2013). The majority of psycholinguistic or phonologically-oriented perceptual studies on gemination are within-language studies of native speakers (Pattani Malay: Abramson, 1986; Kelantan Malay: Hamzah, 2013; Cypriot Greek: Muller, 2001; Swiss German: Kraehenmann, 2001; Tashlhiyt Berber: Ridouane and Hallé, 2010). The situation is similar for vowel quantity contrasts, with most of the cross-linguistic studies on native vs. second language learners of languages with contrasting vowel quantity such as Japanese, Swedish, Finnish, etc. (e.g., McAllister et al., 2002; Hirata, 2004; Ylinen et al., 2005), Note that some studies investigated both vowel and consonant quantity. In particular, one large cross-linguistic study investigated the interaction between vowel and consonant duration in the perception of consonant quantity (Kingston et al., 2009).

This paper contributes to the study of nonnative perception of *consonant* quantity contrasts by examining *naïve* listeners rather than L2 learners. We explore how Berber-naïve French listeners, for whom all segmental quantity contrasts are, in principle, phonemically nonnative, discriminate a particular set of Tashlhiyt Berber (henceforth, Tashlhiyt) singleton-geminate consonant contrasts. We begin with (1) a brief sketch of the state of affairs with respect to the implementation of segmental quantity in French, and (2) a brief review of a few previous studies that have explored French listeners' perception of either native or nonnative quantity contrasts.

### Segmental quantity in french

French has no duration quantity contrasts in its phonemic repertoire, whether for consonants (geminate vs. singleton consonants) or vowels (long vs. short vowels). That is, there are, in principle, no minimal pairs of French words that would differ purely in consonant or vowel duration. However, quantity contrasts *do* occur at the margins of lexical phonology: this is the case of “fake” geminates. Geminate consonants or vowels may appear across word or morpheme boundaries in French just as in English, German, and many other languages, as in (1a–b). Geminate consonants may also result from schwa deletion, as in (2). Finally, gemination of the /r/ consonant is also observed in inflected verb forms, as in (3). This latter case may seem to produce “true” lexical minimal pairs, but it should rather be viewed as a case of vowel-deletion: *courrai* < ^*^courirai < *courir* (cf. *finirai* < *finir*). Diachronically, these forms derive from the deletion of atonic vowels in Latin (e.g., *je mourrai*/ mur.rε/ < mor(i)raio < morīre habeo). Similar forms obtain, synchronically, from schwa deletion in –*er* verbs whose stem ends with *r* (e.g., *il déclarerait* /deklar.rε/ < /deklarərε/; see Meisenburg, 2006, for more examples).

(1a) *il frappe pas* /frap.pa/ ‘he doesn't hit’ vs. *il frappa* /fra.pa/ ‘he hit’
(1b) à apprendre /aapr$\tilde{\alpha }$dr/ ‘to be learned’ vs. à prendre /apr$\tilde{\alpha }$dr/ ‘to be taken’
(2) *là dedans* /lad.d$\tilde{\alpha }$/ ‘in there’ vs. *la dent* /la.d$\tilde{\alpha }$/ ‘the tooth’
(3) *il courrait* /kur.rε/ ‘he would run’ vs. *il courait* /ku.rε/ ‘he was running’

Whereas, (1–3) show examples of geminate-singleton minimal pairs at the sentence level, there are many other occurrences of geminate consonant utterances that are not minimal pairs, as in (4). Geminate consonants thus do occur in French. In most cases, two identical consonants are brought into contact, or concatenated, due to word juxtaposition or to schwa deletion. In other words, French geminates are concatenation, or “fake,” geminates (Hayes, 1986; Schein and Steriade, 1986).

(4) *robe beige* /rɔb.bεӡ/ ‘beige dress’; *netteté* /nεt.te/ </nεtəte/ ‘sharpness’

As for vowels, standard French (as spoken in the Île de France area) has no vowel quantity contrast, except simple vs. concatenated vowels at the sentence level as in (1b). French has been argued to have instead a vowel tense-lax contrast (Jakobson et al., 1952; Tranel, 1987), i.e., marked by vowel quality as well as duration differences. Yet, some regional varieties of French have maintained a final vowel duration contrast to mark grammatical gender, as in *ami*-*amie* (“boyfriend” vs. “girlfriend”) pronounced [ami]-[ami:]. This is the case of French spoken in Switzerland and Belgium but not of, for example, Parisian French.

Given these properties of standard French, we may ask whether Parisian French listeners can distinguish between short and long vowels and between short and long consonants.

### French perception of segmental quantity

For vowels, Duncan (1975) manipulated vowel duration of naturally spoken French words and showed that French listeners (of mixed regional origin) were sensitive to vowel duration for pairs such as *mettre*-*maître* (“to put” vs. “master”) in which /ε/ may be pronounced longer in *maître* than *mettre*. She found that a 32 ms lengthening of /ε/ in *mettre* was sufficient to lower the initially dominant rate of “mettre” judgments down to 50% (More detailed quantitative data were unfortunately not reported in her study). Grosjean et al. (2007) found that Parisian French listeners did quite poorly at identifying words ending with a long vowel (such as *amie* [ami:] as opposed to *ami* [ami]) compared to Swiss French listeners who performed at near ceiling level. As for perception of vowel quantity contrasts in languages other than French, Parisian French listeners have been shown to discriminate Japanese vowel quantity contrasts less well than native Japanese listeners (Dupoux et al., 1999). However, the Parisian French subjects in the Dupoux et al.'s (1999) study nonetheless achieved rather high-level performance: from 80 to 90% correct, depending on the experimental condition.

For consonants, the literature is rather scarce on French sensitivity to consonant duration. Delattre (1971a,b) examined the production and perception of fake and true geminates in English, German, Spanish, and French. For example, he compared noun phrase pairs such as *la ville limite* /lavil.limit/ (“the extreme town”) vs. *la vie limite* /lavilimit/ (“the extreme life”) (French), or word pairs in Spanish such as *pero* (“but”) vs. *perro* (“dog”) (Spanish). He found that consonant duration was the main cue to gemination for both production and perception, with intensity changes and preceding vowel duration as secondary cues. Interestingly, on the basis of intensity curves compared for geminate vs. singleton consonants, Delattre proposed that gemination involved a double articulation, or a “rearticulation,” an idea followed up by Lehiste et al. (1973), who used electromyographic data to conclude that both phonemic and concatenation geminates are produced with a double articulation in Estonian and in English. The perceptual data reported in Delattre (1971b) unfortunately was mostly qualitative. Delattre synthesized continua between minimal pairs of French utterances (e.g., between *elle aime* /εlεm/ ‘she loves’ and *elle l'aime* /εl.lεm/ ‘she loves her/him’) by varying the duration of the critical consonant (/l/, /n/, or /s/). This is a crucial manipulation, as we will see later, because prosodic factors other than duration were kept constant. He ran French, English, German, and Spanish listeners on these stimuli, using a 2AFC identification test. According to Delattre, the non-French listeners “understood enough French to distinguish between simple pairs of utterances, yet …had not lost their native habits of speaking and hearing”. In Delattre's report of the results, “the geminates were separated from the single consonants by a wide range of ambiguous durations” (Delattre, 1971b, p. 100), presumably meaning rather low categorization precision (i.e., shallow slope at categorical boundary). Delattre noted slight differences between listener groups. For example, German and Spanish subjects switched from single to geminate consonant categorization of /s/ at shorter durations than English and French subjects. Delattre also found that “the duration of the preceding vowel was not a factor in the perception of consonant gemination” (Delattre, 1971b, p. 112). This conclusion, which is contrary to that of Kingston et al. (2009), was however based on production data, not perception data. Delattre's conclusion that duration is the main factor for the *perception* of consonant gemination therefore remains unwarranted.

Since these early efforts to examine the perception of fake geminate consonants, by French listeners among others, we are only aware of one more recent study by Meisenburg (2006). Meisenburg, just like Delattre (1971a,b), looked at the production and perception of a few fake gemination minimal pairs, namely, *frappe pas* /frap.pa/ vs. *frappa* /fra.pa/, *courrait* /kur.rε/ vs. *courait* /ku.rε/ [examples (1a) and (3) above], and *il l'a dit* /il.ladi/ ‘he said it’ vs. *il a dit* /i.ladi/ ‘he said.’ Each stimulus was produced once by 12 French speakers, instructed to avoid prosodic marking, and presented to 16 native French listeners, who were administered a 2AFC forced choice identification task. In Table 1, we summarize the production (segment duration) and perception (response accuracy) data reported in more detail in Meisenburg (2006). We added a correlation measure between segment duration and response accuracy (“D × A” correlation): This correlation is positive for geminate and negative for singleton consonants, except in *courait*. The correlation strength measures the importance of segment duration in the singleton-geminate judgments made by the subjects. As can be seen in Table 1, the identification performance was about 80% for two contrasts out of three. The performance was lowest for *courait-courrait*.

**Table 1:** **Meisenburg's (2006) data**.

	***frappa***	***frappe pas***	***courait***	***courrait***	***il a dit***	***il l'a dit***
Segment duration (ms)	91	174	44	75	47	104
Correct identification	75%	86%	94%	40%	78%	83%
D × A correlation	−0.98	0.56	0.05	0.92	−0.81	0.68

For each of three minimal pairs, duration of the critical segment /p/, /r/, or /l/, accuracy (correct identification), and duration × accuracy correlation (D × A).

To summarize the available data on the French listeners' perception of speech segment quantity in French (and in Japanese for vowels), it seems well above the chance level most of the time, for both consonant and vowel quantity, although vowel quantity may be more difficult than consonant quantity since it does not convey a linguistic distinction, at least in the standard, Parisian variety of French.

Thus far, we have reviewed data bearing directly on the perception by French listeners of duration quantity distinctions. Other data suggest that French listeners are sensitive to subphonemic consonant duration differences distinguishing, for example, plain consonants and liaison consonants (Spinelli et al., 2003, also see Spinelli et al., 2007, for similar findings with French elision; (Snoeren et al., 2008), for plain vs. assimilated consonants). In those cases, subphonemic duration differences are very modest (e.g., liaison /r/ was found to be 59–64 ms long as compared to 71 ms for plain onset /r/ in Spinelli et al., 2003). Yet, they result in differential associative semantic priming such that listeners tend to recover the speaker's intended meaning.

In this study, we focus on the French perception of consonant quantity contrasts at a *pre-lexical level of perception*, using for that purpose a discrimination task on nonnative quantity contrasts. We compare different types of singleton-geminate contrasts in terms of consonant type and within-utterance position, as explained in more detail in the following. For all contrast types, performance of native listeners is taken as the reference for optimal perception, against which the French performance can be compared.

### The present study

We cited in the Introduction Section a within-language study by Ridouane and Hallé (2010), which tested Tashlhiyt listeners on contrasts between singleton and geminate consonants in Tashlhiyt word minimal pairs. They found near ceiling Tashlhiyt performance for word-initial voiced stop and fricative as well as for word-final voiceless stop contrasts, but rather poor performance on word-initial voiceless-stop contrasts (between 55 and 70% correct discrimination). The Tashlhiyt listeners' performance only slightly improved with audio-visual stimuli. Unpublished data collected for French listeners' discrimination on these same voiceless-stop stimuli showed that French performance was only slightly poorer than for Tashlhiyt listeners. These contrasts are presumably difficult since, as shown in Ridouane (2007), Tashlhiyt utterance-initial geminate and singleton voiceless stops do not reliably differ acoustically, although they clearly differ in their articulation as reflected in electropalatographic measurements of tongue-to-palate contact: in particular, the durations of their closure, which cannot be perceived word-initially from the acoustic signal, are in a ratio close to 2:1. These word-initial voiceless stop contrasts therefore would not allow a classic cross-linguistic comparison between native control listeners performing near ceiling on native contrasts and French listeners expected to perform more poorly on nonnative contrasts.

We therefore chose for the present study the easier Tashlhiyt singleton-geminate contrasts, on which native listeners have been reported to perform near ceiling (Ridouane and Hallé, 2010). These contrasts are substantiated by clear acoustic differences in *duration*: for example, differences in frication duration for fricatives, or in pre-release voiced murmur duration for voiced stops. In this paper, we thus focus on these easier contrasts and examine their perception by French vs. Tashlhiyt listeners. One reason to use Tashlhiyt as the target language (other than the fact that French listeners are unlikely to have been exposed to Tashlhiyt) is that singleton-geminate contrasts in this language are almost exclusively marked by *duration* differences (Ridouane, 2007), which are directly relevant to quantity distinctions. Although the primary acoustic cue to gemination is always duration (Lahiri and Hankamer, 1988; Hankamer et al., 1989; Ridouane, 2010), there are some languages in which other phonetic or prosodic cues participate in the distinction between geminate and single consonants: for example, accentual cues in Pattani Malay (Abramson, 1986), VOT differences in Cypriot Greek (Armosti, 2009), following vowel quality differences in Japanese (Kawahara, 2006). Because we address here the issue of the perception of nonnative consonant duration quantity contrasts, a target language such as Tashlhiyt, for which the possible confounds with other dimensions than *duration* are minimized, is highly desirable. The rather good French performance on fake geminate consonants reported above (Delattre, 1971b; Meisenburg, 2006), at least on /p:/, /l:/, /n:/, and /s:/, which is presumably due solely to durational differences, might predict rather good performance by French listeners on the Tashlhiyt data as well.

We chose Tashlhiyt contrasts that cover a rather wide spectrum of obstruents in terms of acoustic intensity. The acoustic substance of their constriction or closure portion varied from silence (word-final voiceless stops: e.g., *fit-fitt*), to low-intensity voicing murmur (word-initial voiced stops: e.g., *bi-bbi*), with strident frication (word-initial fricatives: e.g., *sir-ssir*) in between. Our initial guess was that singleton-geminate discrimination would be easier when carried by higher-intensity portions of acoustic signal. More precisely, we predicted that, whereas native speakers of Tashlhiyt likely perform near ceiling on all these contrasts, French listeners should encounter the greatest difficulty with voiceless stops (silence) and the least difficulty with voiceless fricatives (strident frication). We will see that this prediction was not borne out and that the acoustic intensity differences among the contrasted consonants was definitely not the sole factor determining nonnative performance.

## Experiment 1

We used natural utterances of Tashlhiyt minimal-pairs as singleton-geminate contrasts in a cross-language AXB discrimination test, comparing native speakers of Tashlhiyt and naive French listeners with no exposure to Tashlhiyt or similar languages. The AXB paradigm was chosen because it taps into a sufficiently abstract level of processing to disclose potential difficulties with nonnative contrasts, without imposing heavy memory load or letting subjects to respond on the basis of low-level auditory-acoustic differences and similarities.

### Methods

#### Participants

Twelve native speakers of French, students or teachers at Paris 3 University, aged 21–57 years (mean 33.4, SD 13 years), and 23 Tashlhiyt native speakers, students at Ibnou Zohr University in Agadir, aged 19–37 years (mean 26.1, SD 4.9 years), volunteered to participate in the experiment. French and Tashlhiyt participants were tested in Paris and Agadir, respectively. None of the 12 French participants had had any exposure to Tashlhiyt or any language using word-initial and word-final geminate-singleton contrasts. None of the French or Tashlhiyt participants reported having any hearing deficits or any kind of language impairment.

#### Stimuli and design

Seven geminate-singleton contrasts were used: three voiced stop contrasts in word-initial position (*bi-bbi, diR-ddiR*, and *gar-ggar*)^1^, two voiceless fricative contrasts in word-initial position (*fit-ffit*, and *sir-ssir*), and two voiceless stop contrasts in word-final position (*fit-fitt*, and *hat-hatt*). The distribution of consonants within the three contrast-types reflects the distribution observed in Tashlhiyt in general (Ridouane, 2014), in order to hold constant the distributional input that native speakers of Tashlhiyt naturally experience. There were thus a total of 14 items. Four repetitions of each item, produced in isolation by a native speaker of Tashlhiyt, were retained as experimental stimuli. In Tashlhiyt, just as in French, voiced stops are realized as phonetically voiced in any position, that is, with a voiced closure portion whose acoustic realization is a voicing murmur (i.e., pre-voicing: voicing prior to stop release). Geminated voiced stops differ from their singleton counterparts essentially by a longer voiced closure portion. Note that the closure duration distinction holds as well for Tashlhiyt voiceless stops, even in absolute initial position (Ridouane, 2007), although of course it is silent rather than voiced. Likewise, for Tashlhiyt fricatives, geminates differ from singletons essentially by a longer constriction duration. Importantly, Ridouane (2007) did not find acoustic or articulatory cues other than duration that reliably distinguishing geminates from singletons in Tashlhiyt. We ran acoustic measurements on the retained stimuli. As expected, the clearest cue to gemination is durational. The critical durations of the stimuli are summarized in Table 2. In all cases, the geminate's closure duration approaches or exceeds twice that of the singleton.

**Table 2:** **Mean durations (ms) of prevoicing, frication, and silent closure durations for the three contrast types (word-initial voiced stops and fricatives, and word-final stops), detailed by contrasts**.

**Contrast**	**Singleton**	**Geminate**
*bi-bbi*	88 (12.8)	215 (10.5)
*diR-ddiR*	65 (11.5)	215 (12.7)
*gar-ggar*	56 (20.4)	202 (16.6)
Mean prevoicing	70 (20.0)	211 (13.9)
*fit-ffit*	124 (26.0)	233 (15.9)
*sir-ssir*	136 (10.0)	269 (20.1)
Mean frication	130 (19.3)	252 (25.5)
*fit-fitt*	69 (7.9)	211 (15.3)
*hat-hatt*	79 (7.9)	218 (19.9)
Mean silent closure	72 (8.0)	211 (16.2)

Standard deviations are shown between parentheses.

The duration differences shown in Table 2 were all significant at the *p* < 0.00001 level, according to two-tailed *t*-tests in which geminates and singletons were compared for each of the three types of contrast [word-initial voiced stops: *t*_(22)_ = 20.05; word-initial fricatives: *t*_(14)_ = 10.79; word-final /t/-/t:/: *t*_(22)_ = 26.65]. They were accompanied by subtler differences, some of which reached significance on two-tailed *t*-tests. For instance, in the /t/ coda series, the longer closure for /t:/ than /t/ was partly compensated by a shorter onset consonant (88 < 111 ms), *t*_(22)_ = 3.99, *p* < 0.001, and initial vowel (104 < 146 ms), *t*_(22)_ = 8.68, *p* < 0.00001. In the voiced stop onset series, both the mean intensity and F0 of the voicing murmur were higher in singleton than geminate consonants [F0: 127 > 118 Hz, *t*_(22)_ = 3.10, *p* < 0.01; intensity: 46.5 > 41.9 dB, *t*_(22)_ = 3.24, *p* < 0.005], that is, they showed a time-intensity trade in production. We also measured the intensity of these voicing murmurs relative to the next vowel (/a/ or /i/), a measure which is more independent of overall speaking loudness: on this measure as well, singletons were found to have higher intensity voicing murmurs than geminates: −16.7 > −21.4 dB relative to the next vowel. The geminate and singleton word-initial fricatives did not differ in terms of mean intensity; they were about 6 dB higher than the voiced murmur in word-initial voiced stops (50.2 vs. 44.2 dB), *t*_(38)_ = 4.44, *p* < 0.0005. Finally, there was a marginal trend for geminate fricatives to have a lower mean Harmonic-to-Noise ratio (HNR) than singleton fricatives (0.8 < 4.0 dB), *t*_(14)_ = 1.91, *p* = 0.074, i.e., to be “noisier.” The same trend was found with the vowel preceding /t:/ compared to /t/ (9.7 < 12.7 dB), *t*_(22)_ = 1.93, *p* = 0.063. That is, in some instances, gemination seemed to be associated with a lower harmonicity measure.

Each contrast was presented four times in each of the four possible AXB combinations (AAB, ABB, BBA, and BAA) so that the stimuli appeared equiprobably in each position within the AXB triplets. There were thus 112 trials for the seven contrasts under scrutiny. These trials were part of a larger design including 128 other trials on word-initial voiceless stop singleton-geminate Tashlhiyt contrasts such as *ks-kss* or *tili-ttili*. The perception of these contrasts by Tashlhiyt listeners were reported in Ridouane and Hallé (2010) and in a forthcoming chapter comparing production and perception data; the French listeners' data on these contrasts will be reported elsewhere. In the present report, we will treat the 128 trials such as *tili–ttili* as filler trials and the other 112 as test trials. The trials were presented in 16 blocks, with one trial for each of the seven test trials in each block. Each block thus contained the same distribution of trials in terms of contrast-types as the entire experimental session, allowing for a time-course analysis of subjects' responses. Both trials within blocks and blocks within the test session were randomized, with a different randomization for each subject. The 16-block test session was preceded by 10 training trials on five contrasts, none of which was used in the test trials: *daR-taR, dar-tar, kijji-gijji, tid-ttid, and jutid-juttid*. Note that only the last two contrasts were geminate-singleton contrasts with /t/-/t:/ in initial vs. medial position.

#### Procedure

Participants were tested individually in a quiet room and received the speech stimuli through professional quality circumaural headphones (Sennheiser HD 518). On each AXB trial, participants were presented with three stimuli and had to indicate whether the second item X was a better category match to the first or the third stimulus, by depressing the response key labeled “1” or “3.” The inter-stimulus (offset to onset), inter-trial, and inter-block intervals were set to 1, 4, and 9 s, respectively. Response times were measured from the end of the critical consonant in the X stimulus. The experiment was run using the DMDX software (Forster and Forster, 2003). Participants received feedback for the training trials (accuracy and response time) but not for the test trials.

### Results

#### Correct discrimination response rate

**Figure 1:**
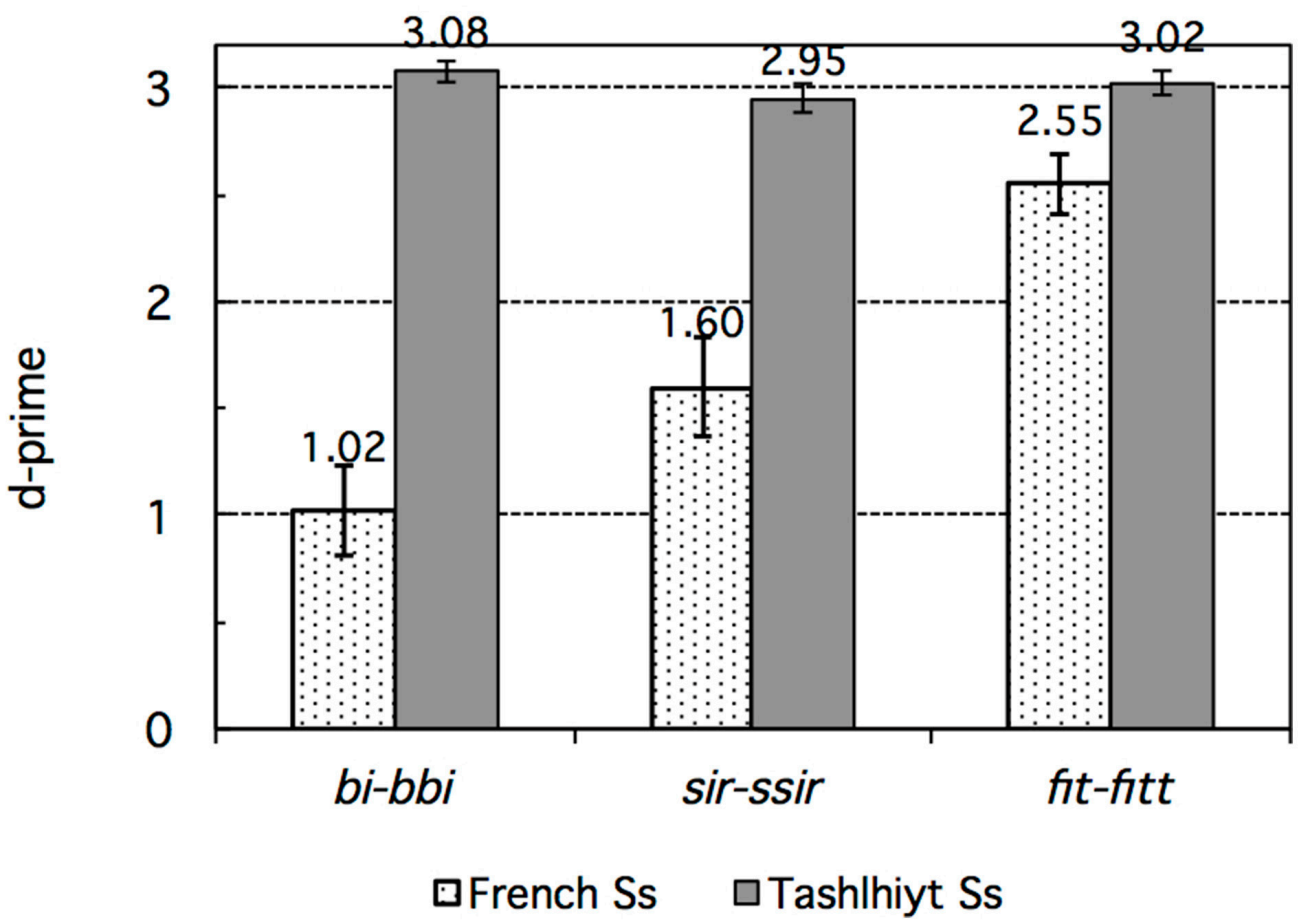
**Experiment 1: French vs. Tashlhiyt participants' d-prime data for the three types of contrasts subsumed as *bi–bbi*, *sir–ssir*, and *fit–fitt***. The error bars represent standard errors.

The Tashlhiyt participants performed near ceiling on all contrasts, as expected. By comparison, the French participants performed poorly. They performed the most poorly on the word-initial voiced stop contrasts (e.g., *bi-bbi*), less poorly on the word-initial voiceless fricative contrasts (e.g., *fit-ffit*), and best on the word-final /t/-/tt/ contrasts (*fit-fitt* and *hat-hatt*). Table 3 shows the accuracy data detailed by contrast. Figure 1 shows the corresponding d-prime data, pooled by contrast-type. We computed d-prime values from the response data in the AXB task following MacMillan and Creelman (2005): for each AXB trial, “A” responses (response key labeled “1”) were (arbitrarily) treated as hits when correct, and as false alarms when incorrect. An analysis of variance was run on the d-prime data, with Subject as the random factor, Language as a between-subject factor (French vs. Tashlhiyt), AXB trial Target (X = singleton vs. geminate), and Contrast type (the three types under scrutiny) as within-subject factors. Note that the AXB trial Pattern, or primacy/recency factor (primacy: X = A vs. recency: X = B), which has been examined in some studies using an AXB procedure (Best et al., 2001; Hallé and Best, 2007), cannot be analyzed in the d-prime data since each d-prime value is computed from accuracy rate on primacy trials and error rate on recency trials. We thus used mixed model log-odds regression analyses, with both subjects and items as random factors, on the raw binary data (correct/incorrect) to examine the Pattern factor, among others. It was not significant, for either the French or the Tashlhiyt subjects' data^2^. We do not report the detail of these analyses because they yielded exactly the same patterns of results as obtained with the analysis of variance on the d-prime data. We now return to this analysis. Let us first discuss the structural factor Target. Target was significant overall, *F*_(1, 33)_ = 20.05, *p* < 0.0001, and strongly interacted with Language, *F*_(1, 33)_ = 16.62, *p* < 0.0005. Indeed, Target did not reach significance in the Tashlhiyt group, *F*_(1, 22)_ = 2.51, *p* = 0.13, whereas it was significant in the French group, *F*_(1, 11)_ = 19.82, *p* < 0.001, with a poorer performance overall when X in AXB was a singleton than geminate stimulus (d-primes: 1.27 < 1.97; corresponding discrimination rates: 70.1 < 80.9%). Therefore, discrimination was more difficult around singleton than geminate stimuli for French but not Tashlhiyt participants. If we reason in terms of prototype-induced “magnet effects” (Kuhl, 1991; Kuhl et al., 2008), this asymmetry suggests that, for French listeners, singleton consonants are more typical of the corresponding native stops and fricatives than are geminates. In contrast, singletons and geminates did not differ in typicality for Tashlhiyt listeners. We turn now to the main factors of interest, Language and Contrast.

**Table 3:** **Experiment 1: Discrimination rate data detailed by contrast (with standard deviations)**.

**Contrast**	**French Ss**	**Tashlhiyt Ss**
	**% Correct (SD)**	**% Correct (SD)**
*bi-bbi*	67.2 (28.5)	97.6 (8.3)
*diR-ddiR*	65.3 (27.4)	97.3 (9.4)
*gar-ggar*	65.6 (29.8)	96.6 (9.7)
*fit-ffit*	73.8 (30.0)	94.8 (13.1)
*sir-ssir*	76.7 (27.4)	95.9 (10.0)
*fit-fitt*	91.3 (13.5)	95.7 (10.9)
*hat-hatt*	88.5 (16.8)	97.3 (7.8)

The main factor Language was highly significant, *F*_(1, 33)_ = 98.69, *p* < 0.00001, reflecting the overall better performance of Tashlhiyt than French participants. The Contrast factor was also significant overall, *F*_(2, 66)_ = 13.53, *p* < 0.00001. The strong Language × Contrast interaction, *F*_(2, 66)_ = 29.59, *p* < 0.00001, suggested that the Contrast effect differed between Tashlhiyt and French participants. Indeed, Contrast was significant only for French, *F*_(2, 22)_ = 18.14, *p* < 0.00001, not Tashlhiyt listeners, *F*_(2, 44)_ = 1.81, *p* = 0.18. As can be seen in Table 3, performance was near ceiling for Tashlhiyt participants for all contrasts, whereas it varied substantially with contrast type for French participants: about 66 < 75 < 90% (d′: 1.02 < 1.60 < 2.55) for the word-initial voiced stop, word-initial fricative, and word-final voiceless stop contrasts, respectively. The corresponding pairwise differences in the d-prime data all were significant [initial voiced stop vs. initial fricative: *F*_(1, 11)_ = 6.90, *p* < 0.05; initial fricative vs. final voiceless stop: *F*_(1, 11)_ = 10.84, *p* < 0.01].

We may note that even the lowest French performance (66% correct discrimination rate and d′ = 1.02 on the word-initial voiced stop contrasts) was above chance (or null sensitivity) level as shown by *t*-test comparisons with the 50% chance level on the accuracy rate data or, equivalently, with d′ = 0 on the d-prime data. However, whereas the Tashlhiyt performance was, as anticipated, optimal and near ceiling level for all three types of contrasts, the French performance was below the Tashlhiyt performance for all three types of contrasts, as shown by paired comparisons between the French and Tashlhiyt d-prime data for each contrast type (voiced stop: 1.02 < 3.08; fricative: 1.60 < 2.95; word-final voiceless stop: 2.55 < 3.02; *p*s < 0.005).

#### Correct discrimination response times

**Figure 2:**
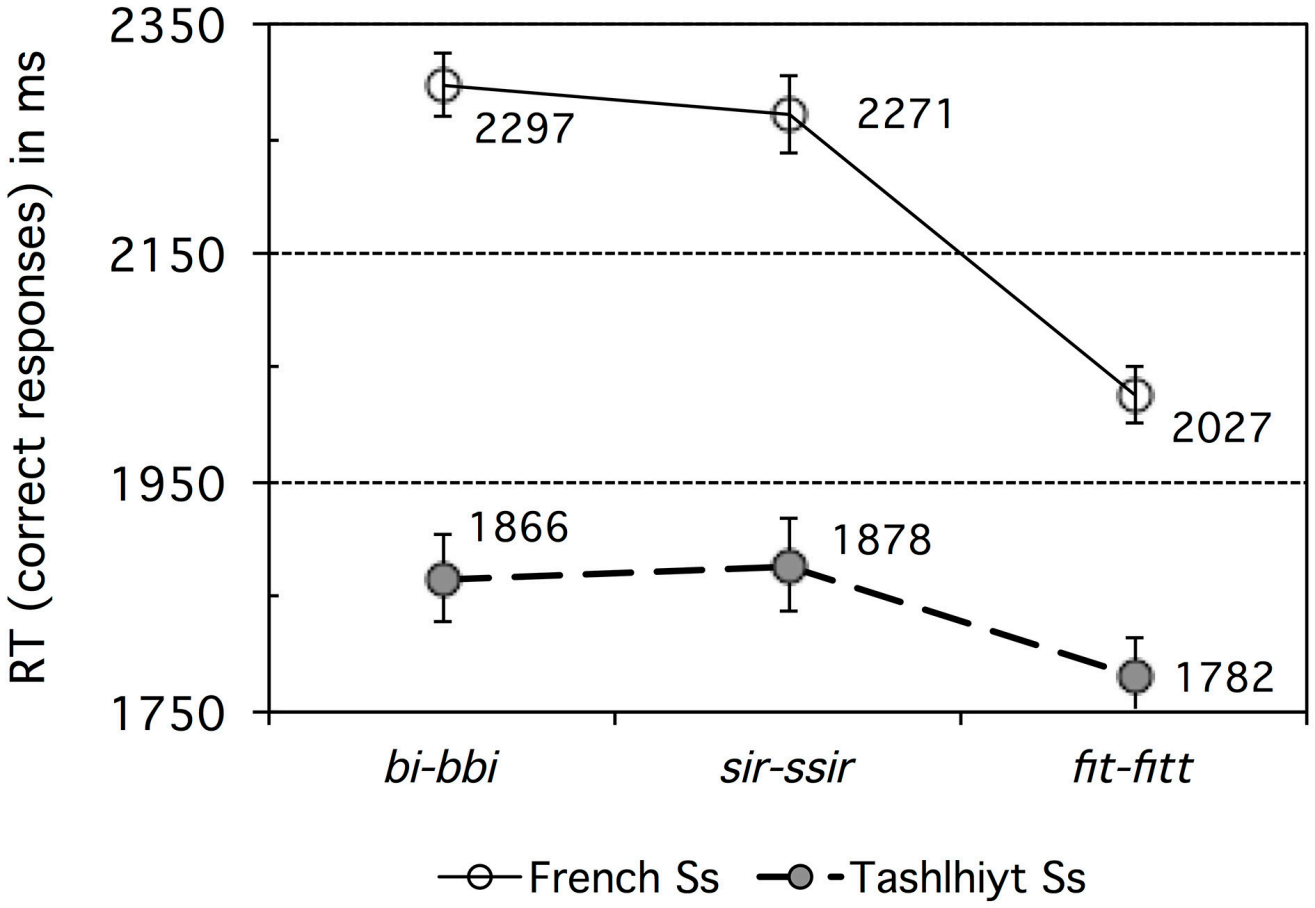
**Experiment 1: French vs. Tashlhiyt participants' response time data for the three types of contrasts subsumed as *bi–bbi*, *sir–ssir*, and *fit–fitt***. The error bars represent standard errors.

Figure 2 shows the RT data for correct responses. Note that RT values were measured from the end of the critical consonant in the second stimulus (X); if the third stimulus (B) had been used instead, RTs would be shorter by 1500 ms in average (based on inter-stimulus interval and average stimulus durations).

The raw RT data was cleaned up by discarding RT values longer than 3.5 s (more than 2 s after stimulus B's reference time) or shorter than 1.5 s (before stimulus B's reference time). About 0.6% of the French RT data was discarded in this way and 0.1% of the Tashlhiyt RT data. An analysis of variance was run on the cleaned-up RT data, using the same factors as for the d-prime data, with the addition of the AXB trial Pattern (or primacy/recency) within-subject factor. This latter structural factor Pattern was significant overall, *F*_(1, 33)_ = 19.80, *p* < 0.0001, but did not interact significantly with Language, *F*_(1, 33)_ = 2.79, *p* = 0.104. Pattern was significant for both groups [French: *F*_(1, 11)_ = 14.13, *p* < 0.005; Tashlhiyt: *F*_(1, 22)_ = 7.31, *p* < 0.05], with shorter RTs for “recency” (X = B) than “primacy” (X = A) trials in both groups (French: 2133 < 2264 ms; Tashlhiyt: 1810 < 1873 ms). That is, X = A responses were generally more difficult than X = B responses. Note that such “recency” effects in AXB discrimination experiments seem to be specifically associated with the subjects' detection of nonlinguistic (as opposed to phonological or phonetic) differences (Crowder, 1971, 1973; Best et al., 2001: 786). Because no parallel recency effects were found in the accuracy data (as analyzed using mixed model log-odds regression: see Section Correct Discrimination Response Rate in Experiment 1), and because the contrasts at stake indeed are phonological for Tashlhiyt listeners, who exhibited clear recency effects for RTs, we must ascribe the recency effects in the RT data to a general bias favoring psychoacoustic detection of nonlinguistic differences. The structural factor Target was significant overall, *F*_(1, 33)_ = 110.59, *p* < 0.00001, and did not interact with Language, *F* < 1. Target was significant for both groups [French: *F*_(1, 11)_ = 26.76, *p* < 0.0005; Tashlhiyt: *F*_(1, 22)_ = 101.19, *p* < 0.00001], reflecting the same pattern: longer RTs for singleton than geminate X targets (French: 2282 > 2114 ms; Tashlhiyt: 1914 > 1769 ms). Therefore, still reasoning in terms of possible “magnet effects,” the RT data suggest that singleton items may be more prototypical than geminate items for both French and Tashlhiyt listeners.

Turning now to the main effects, Language was highly significant, *F*_(1, 33)_ = 13.36, *p* < 0.001, with Tashlhiyt listeners faster than French listeners by about 356 ms. The Tashlhiyt advantage held for all three types of contrast, *p*s < 0.01. However, as suggested by a significant Language x Contrast interaction, *F*_(2, 66)_ = 11.07, *p* < 0.0001, this advantage varied across contrasts: As can be seen in Figure 2, it was the smallest for the word-final voiceless stop contrasts. The Contrast factor was significant overall and for both groups [French: *F*_(2, 22)_ = 53.76, *p* < 0.00001; Tashlhiyt: *F*_(2, 44)_ = 8.25, *p* < 0.001]. For both groups, there was no significant RT difference between the word-initial voiced stop and voiceless fricative contrasts (shorthand: *bi-bbi* and *sir-ssir*, respectively), *F* < 1, and these *bi-bbi* and *sir-ssir* types of contrast yielded longer RTs than the word-final voiceless stop *fit-fitt* type of contrast (French: *p*s < 0.00001; Tashlhiyt: *p*s < 0.01). The significant Language x Contrast interaction (see above) also indicates that the RT advantage for *fit-fitt* over *bi-bbi* or *sir-ssir* was larger for French than Tashlhiyt subjects (256 vs. 91 ms).

To sum up, the RT data largely paralleled the accuracy or d-prime data. For French participants, the *fit–fitt* type contrasts were responded to faster than the other two types, confirming they are easier. For Tashlhiyt participants, accuracy was near ceiling level for all three types of contrasts (cf. Table 3), with some differences in response times, namely faster RTs for the *fit–fitt* type than the *bi–bbi* or *sir-ssir* types.

### Discussion

The results of this discrimination experiment showed that, whereas Tashlhiyt listeners performed at near ceiling level on each of the three types of contrasts, French listeners encountered substantial difficulty with the *bi–bbi* and, to a lesser extent, with the *sir–ssir* type of contrast^3^. Based on both the accuracy or d-prime and the response time data, they encountered considerably less difficulty with the *fit-fitt* type of contrast, for which their performance approached the Tashlhiyt performance, although still significantly well below it. In other words, the data suggest a clear ordering of the Tashlhiyt contrasts in terms of their difficulty for French listeners: *bi–bbi*<*sir–ssir*<*fit–fitt*. This ordering is clearly at odds with the ordering we predicted based on the intensity of the acoustic substance of the consonants involved. However, before dismissing this simple prediction, possible confounds must be examined.

**Figure 3:**
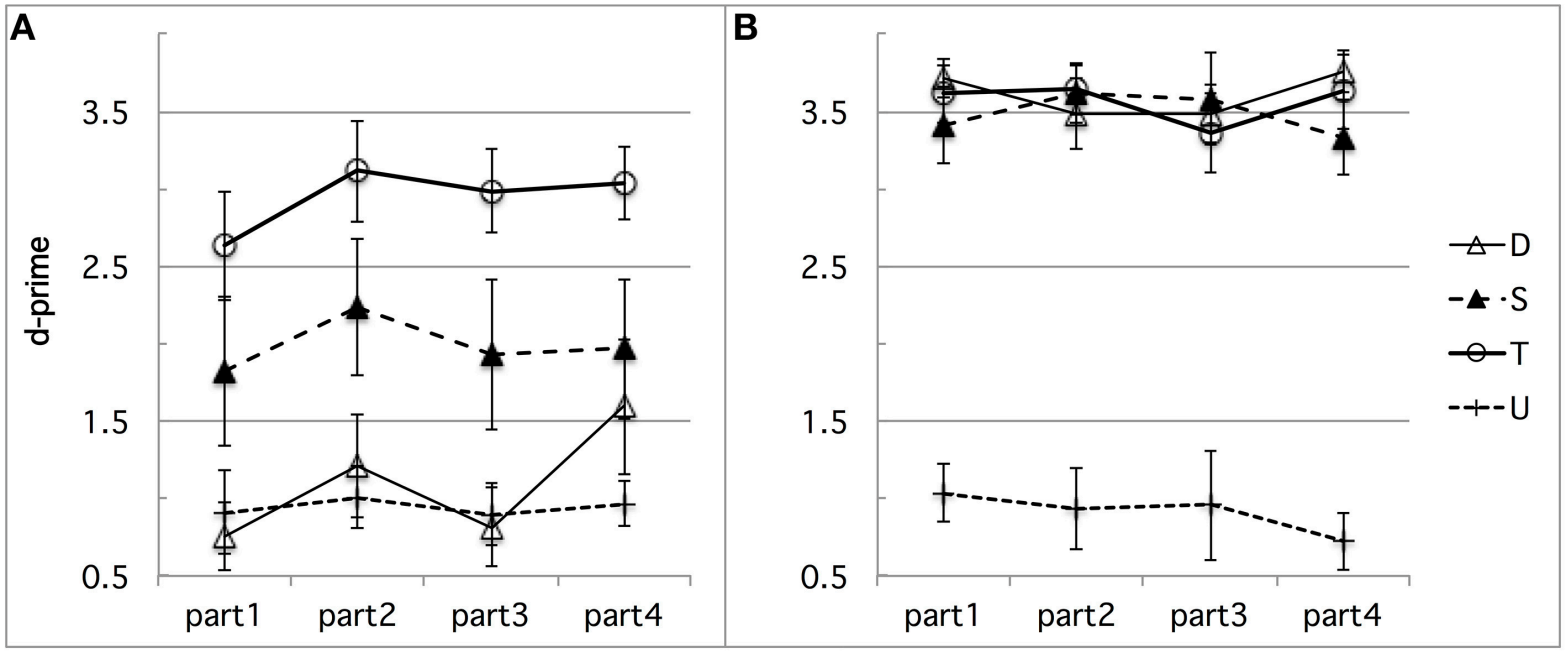
**(A,B)** Experiment 1: **(A)** French and **(B)** Tashlhiyt participants' d-prime data for the three types of test contrasts (D, S, and T for the *bi–bbi, sir–ssir*, and *fit–fitt* contrast types, respectively), in four successive parts of the experimental session. The data on the filler contrasts such as *ks-kks* (noted U) are shown for sake of comparison. The error bars represent standard errors.

**Figure 4:**
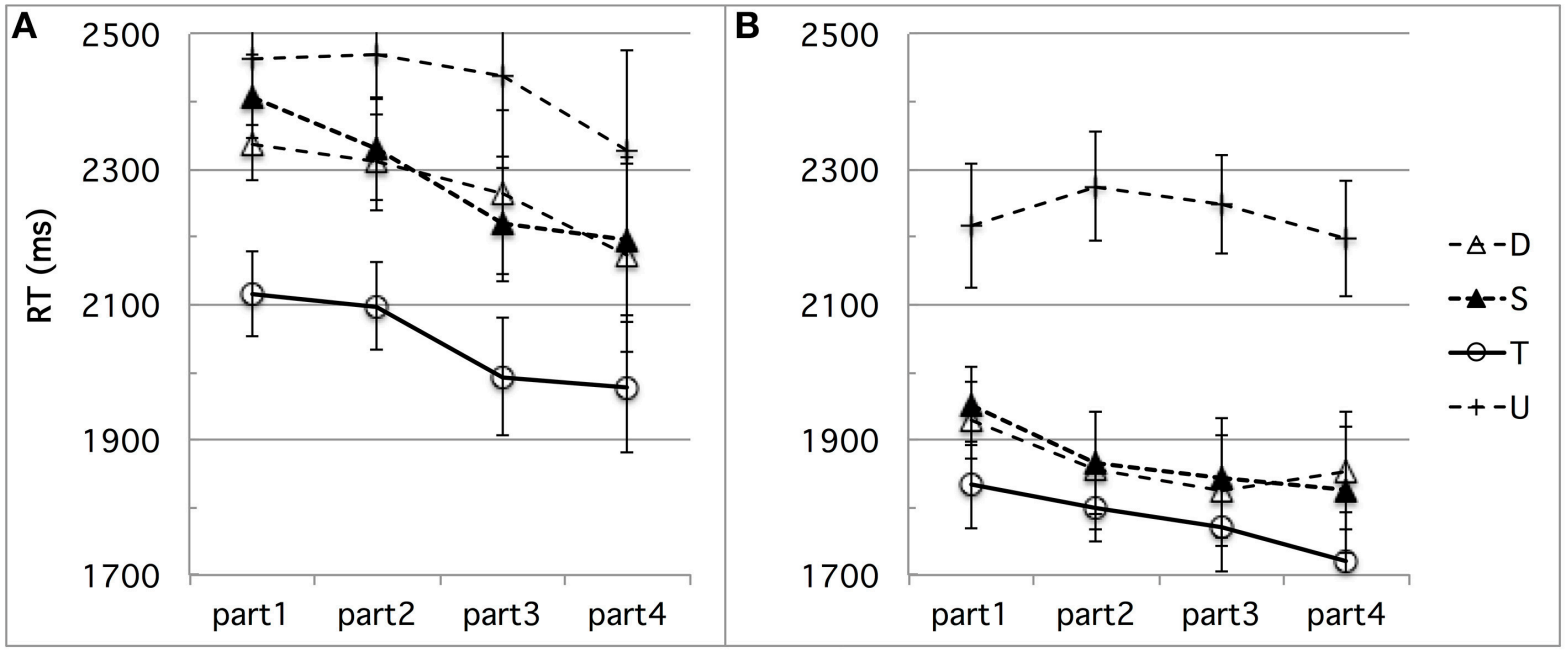
**(A,B)** Experiment 1: **(A)** French and **(B)** Tashlhiyt participants' RT data for the three types of test contrasts (D, S, and T for the *bi–bbi, sir–ssir*, and *fit–fitt* contrast types, respectively), in four successive parts of the experimental session. The data on the filler contrasts such as *ks-kks* (noted U) are shown for sake of comparison. The error bars represent standard errors.

First, learning may have occurred during the experimental session, differently biasing French performance in the direction we found. For instance, French subjects' highest performance on the *fit–fitt* contrast type might be due to the higher incidence of word-final /t/s, singleton or geminate, than any word-initial consonant. There were indeed twice as many word-final /t/s as word-initial /b/s, /d/s, /g/s, /f/s, or /s/s. To test for any kind of learning effect possibly due to the somewhat unbalanced design we used in terms of critical consonants or number of items per contrast type, we conducted a time course analysis of the French discrimination data. Time-course analysis was facilitated by the experimental design into blocks of equal size, each containing the same distribution of trials in terms of contrast types, as explained above in Section Stimuli and Design in Experiment 1. The analysis suggests that some learning took place, resulting in a slight improvement especially for speed, as can be seen in Figure 3 (d-prime data) and Figure 4 (RT data), which show the time-course of performance during the experimental session (test phase) divided into four successive parts^4^. For sake of exhaustiveness, these figures show the performance of both French (A) and Tashlhiyt (B) subjects, and on both the contrasts targeted in this study and the word-initial voiceless stop contrasts, which we treated as fillers in the present study. The analyses of variance we ran, however, were restricted to the French data on non-filler stimuli, with Subject as a random factor, Contrast type (*bi–bbi, sir–ssir*, and *fit–fitt* types) and session Part (parts 1–4) as within-factors, d-prime or RT as the dependent variable. The Part factor was not significant for d-prime, *F*_(3, 33)_ = 1.39, *p* = 0.26, and marginally significant for RT, *F*_(3, 33)_ = 2.63, *p* = 0.066, indicating a trend toward acceleration of the responses. As can be seen in Figure 3A, evidence for learning in terms of d-prime was found only in the case of the *bi-bbi* contrasts in the last part of the session, *F*_(1, 11)_ = 5.01, *p* < 0.05. Importantly, the interaction between Contrast and Part was far from significant for both d-prime and RT, *F*s < 1. That is, the differences among the three types of contrast reported in Section Results in Experiment 1 for the entire session held for each chronological subpart. Or, put another way, there was no sign of differential learning effects according to contrast type that could explain the differences across contrast types, in particular, the best performance for the *fit–fitt* contrast type. This suggests that listeners may tend to learn contrasts across the experimental session on the basis of entire syllables rather than single consonants: For example, *hat–hatt* and *fit–fitt* do not help each other and provide better learning of word-final /t/ than learning of, say, /b/, which only appears in *bi–bbi*.

Another possible confound is that, whereas the intensity of the critical acoustic substance was rather constant within the *sir–ssir* and *fit-fitt* types of contrast (*sir–ssir*: 49.7 vs. 50.7 dB mean intensities, |*t*| < 1; *fit–fitt*: acoustic silence for both singleton and geminate voiceless stop occlusions), it was clearly softer for geminates than singletons in the voiced occlusions of the *bi–bbi* type of contrast: 41.9 < 46.5 dB, *t*_(22)_ = 3.24, *p* < 0.005. Thus, intensity and duration of the voiced occlusion tended to trade off in the acoustic *production* of the *bi–bbi* type of contrasts (cf. the well-known time-intensity trade-off). This intensity-duration trade-off might explain the particular difficulty encountered by French listeners on the *bi–bbi* type of contrasts as a general auditory phenomenon (Shinn, 2007). To test for this possibility, we ran French participants on a second discrimination experiment comprising the same seven contrasts used in Experiment 1, with the addition of a manipulated *bi–bbi* type of contrast, in which the intensity of the voiced occlusions were equalized between singleton and geminate stops. The purpose of this manipulation was to test the impact on French performance of the intensity-duration trade-off in voiced occlusions.

## Experiment 2

This experiment was designed to replicate Experiment 1 with additional contrasts of the *bi-bbi* type, in which signal intensity during prevoicing was manipulated so that geminate and singleton voiced stops were equalized in terms of prevoicing loudness. This manipulation was intended to test for the possibility that the poor French performance on the voiced stop contrasts was due to the prevoicing intensity-duration trade-off found in the original stimuli. Only French participants were tested since Tashlhiyt listeners were already shown to perform near ceiling level.

### Methods

#### Participants

Sixteen native speakers of French, students or teachers at Paris 3 University, aged 21 to 62 years (mean 32, SD 12.5 years) volunteered to participate in the experiment. None of the 16 participants had had any exposure to Tashlhiyt or any language using word-initial geminate-singleton contrasts. None of them reported hearing deficits or any kind of language impairment.

#### Stimuli and design

The same seven contrasts as in Experiment 1 were used (*bi–bbi, diR–ddiR, and gar–ggar; fit–ffit and sir–ssir*; *fit–fitt*, and *hat–hatt*). We added modified versions of the three voiced stop contrasts. There were thus a total of 10 contrasts, i.e., 20 items, with four tokens per item, as in Experiment 1. No filler contrasts were used. The modification consisted in uniformly lowering prevoicing intensity in singleton stops and raising it in geminate stops by about 2 dB, while keeping intact the remaining portion of the stimuli, so that they did not differ any more with respect to the intensity of the voiced occlusion. As a result, the intensity of the modified voicing murmurs relative to the next vowel /a/ or /i/, which was not modified, was about –19 dB for both singleton- and geminate-initial stimuli. The duration of the voicing murmurs were left unchanged (see Table 2). The same training trials as in Experiment 1 were used. Contrasts such as *bi-bbi*, original or modified, were presented each in 16 different trials and the others (*sir-ssir* and *fit-fitt*) each in 32 different trials, in order to maintain the distribution of trials across contrast types of Experiment 1.

#### Procedure

The same procedure as in Experiment 1 was followed, including feedback in training trials.

### Results

#### Correct discrimination response rate

**Figure 5:**
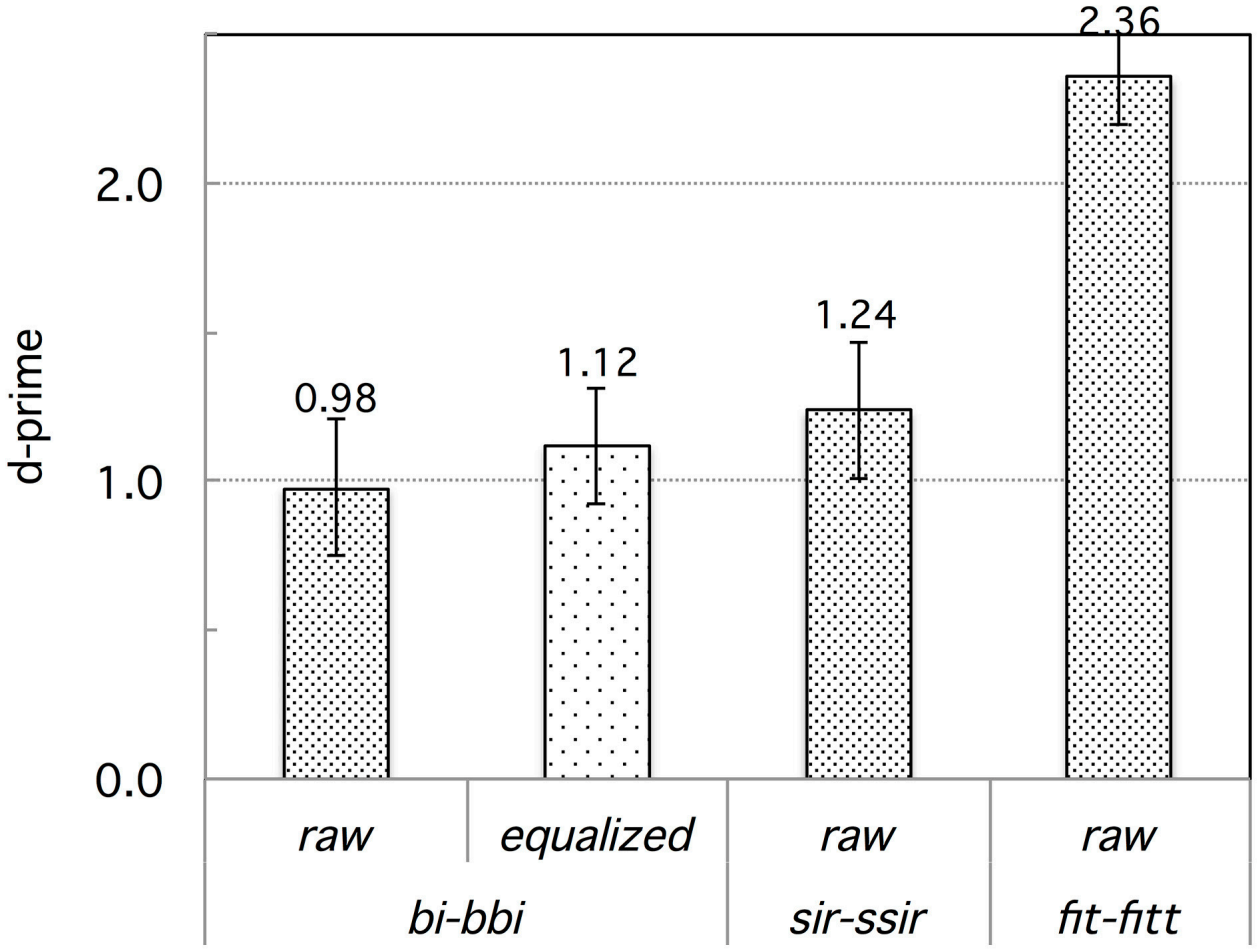
**Experiment 2: French participants' d-prime data for the four types of contrasts subsumed as *bi–bbi* (raw vs. equalized), *sir–ssir*, and *fit–fitt* (raw)**. The error bars represent standard errors.

As in Experiment 1, French participants performed best on the *fit-fitt* contrast type and most poorly on the *bi-bbi* contrast type for either the original or the modified stimuli; they performed only slightly better on the *fit-ffit* than *bi-bbi* contrast type. Table 4 shows the accuracy data (correct discrimination rates) detailed by contrast. Figure 5 shows the corresponding d-prime data, pooled by contrast-type. As in Experiment 1, we ran an analysis of variance on the d-prime data, with Subject as the random factor, AXB trial Target (X = singleton vs. geminate), and Contrast type (with this time four types) as within-subject factors. As in Experiment 1, we used a mixed model log-odds regression analysis to examine the AXB trial Pattern factor (primacy/recency): it was significant, reflecting poorer performance for X = B (recency) than X = A (primacy) trials (66.7 < 72.9 % correct discrimination).

**Table 4:** **Experiment 2: Discrimination rate data detailed by contrast (with standard deviations)**.

**Contrast**	**Original stimuli**	**Prevoicing-modified**
	**% Correct (SD)**	**% Correct (SD)**
*bi-bbi*	67.3 (26.4)	68.0 (26.1)
*diR-ddiR*	59.1 (29.2)	60.7 (29.4)
*gar-ggar*	63.7 (27.7)	65.0 (26.2)
*fit-ffit*	69.2 (22.1)	
*sir-ssir*	67.2 (28.1)	
*fit-fitt*	84.1 (15.7)	
*hat-hatt*	82.4 (18.4)	

Turning back to the analysis of variance of the d-prime data, with the factors listed above, the structural factor Target was significant overall, *F*_(1, 15)_ = 18.90, *p* < 0.001, with better performance for geminate than singleton X targets (% correct: 74.0 > 65.7; d′: 1.73 > 1.12), as in Experiment 1. Again, we may understand these data in terms of typicality: singleton consonants are more typical than geminates for French listeners. We now turn to the main factor of interest, Contrast.

Figure 5 shows an ordering of the contrasts in terms of French performance similar to that found in Experiment 1: *bi-bbi* (original or modified) < *sir-ssir*<*fit-fitt*. However, there was little difference between the three word-initial contrast types and indeed, pairwise comparisons fail to show any significant difference in performance among them, *p*s > 0.23. French participants performed more poorly on these contrasts than on the word-final voiceless stop contrast type, *p*s < 0.001.

#### Correct discrimination response times

**Figure 6:**
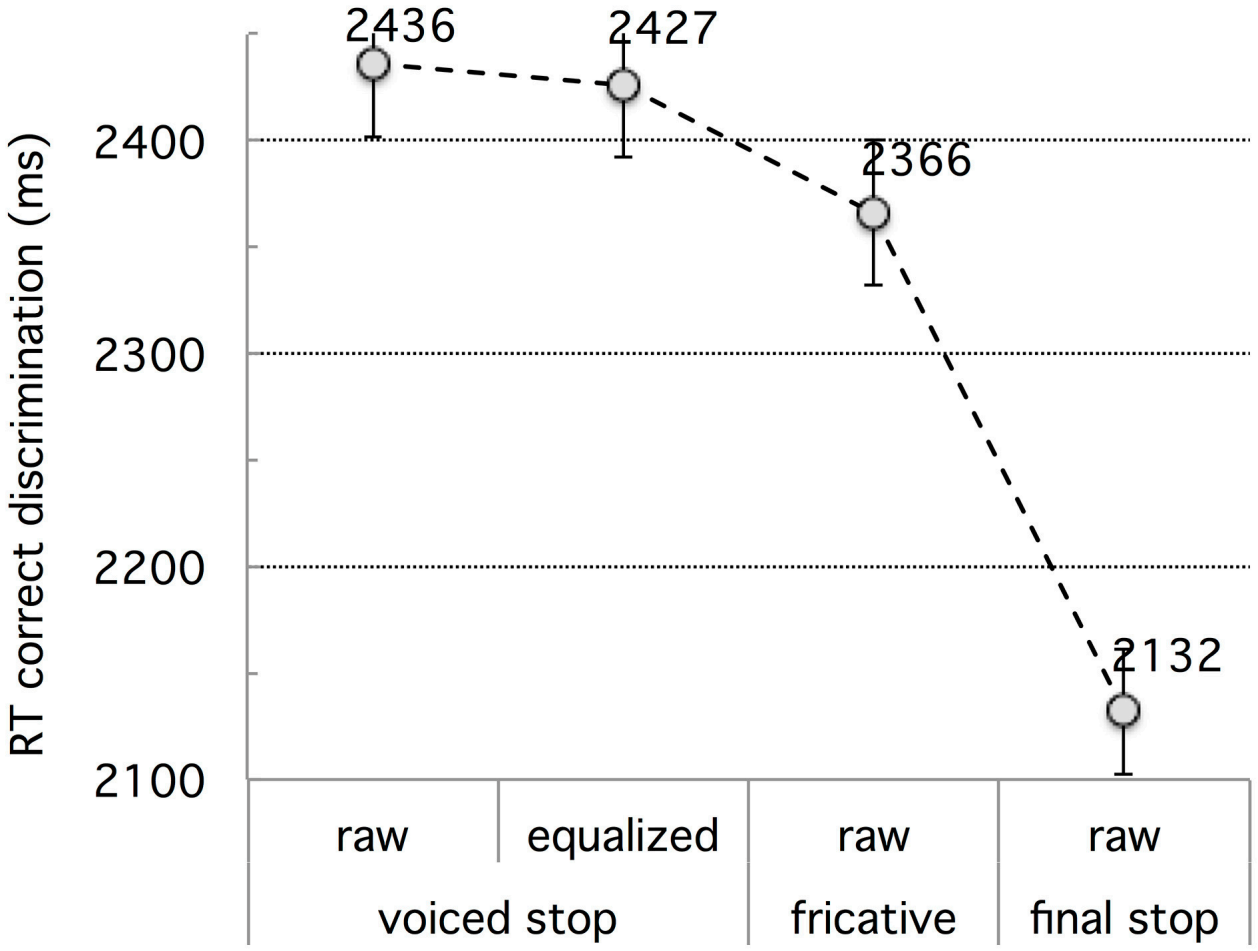
**Experiment 2: French participants' RT data for the four types of contrasts subsumed as *bi–bbi* (raw vs. equalized), *sir–ssir*, and *fit–fitt* (raw)**. The error bars represent standard errors.

Figure 6 shows the RT data for correct responses. RT values were measured and cleaned up in the same way as in Experiment 1, discarding 1.7% of the data. An analysis of variance was run on the cleaned-up RT data, using the same factors as for the d-prime data, with the addition of the AXB trial Pattern (primacy vs. recency) within-subject factor. This latter structural factor did not reach significance. The structural factor Target was significant overall, *F*_(1, 15)_ = 271.09, *p* < 0.00001, with shorter RTs for geminate than singleton X targets (2250 < 2431 ms), as in Experiment 1, suggesting again that singleton items were more prototypical than geminate items for French participants.

The main effect of Contrast was significant, *F*_(3, 45)_ = 49.08, *p* < 0.00001. RTs were the longest for the *bi-bbi* types of contrast with no difference between the original (2436 ms) and equalized (2427 ms) versions, *F* < 1: longer than for the *sir-ssir* (2366 ms) or *fit-fitt* (2132 ms) types of contrasts, *p*s < 0.01; RTs were by far shorter for *fit-fitt* than *sir-ssir, F*_(1, 15)_ = 60.50, *p* < 0.00001. RTs were thus ordered as *bi-bbi* > *sir-ssir* > *fit-fitt*, with a large advantage for the latter type of contrast.

To sum up, the RT data largely paralleled the accuracy or d-prime data. French participants responded faster to the *fit–fitt* type contrasts than the other types, confirming these contrasts are the easiest for them.

#### Time course analyses

**Figure 7:**
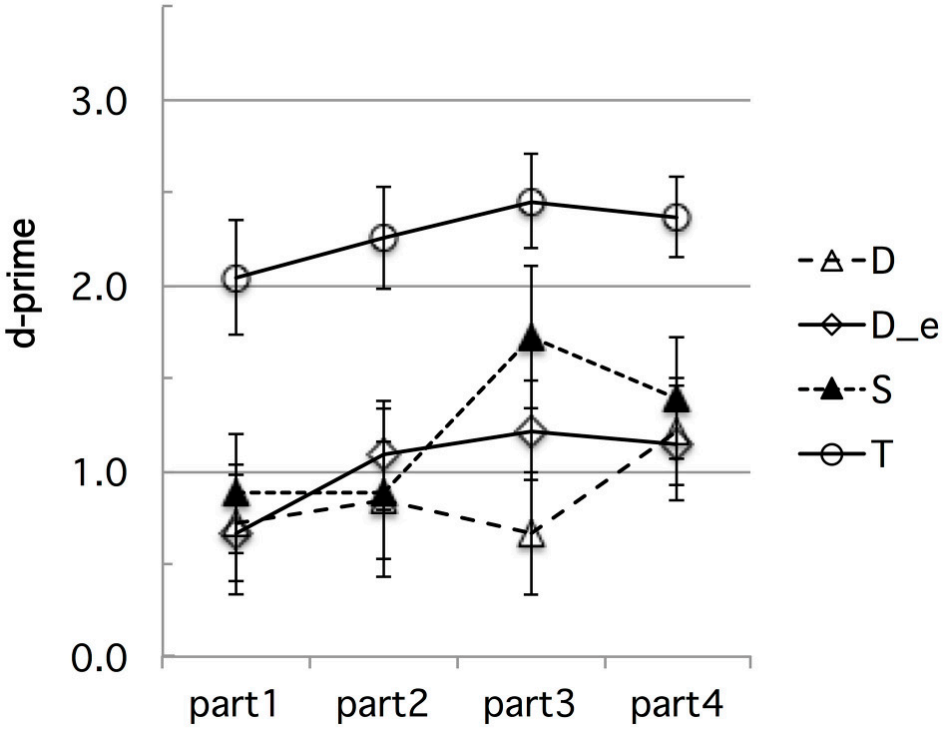
**Experiment 2: French participants' d-prime data for the four types of contrasts (D, D_e, S, and T for the *bi–bbi raw*, *bi–bbi equalized, sir–ssir*, and *fit–fitt* contrast types, respectively), in four successive parts of the experimental session**. The error bars represent standard errors.

**Figure 8:**
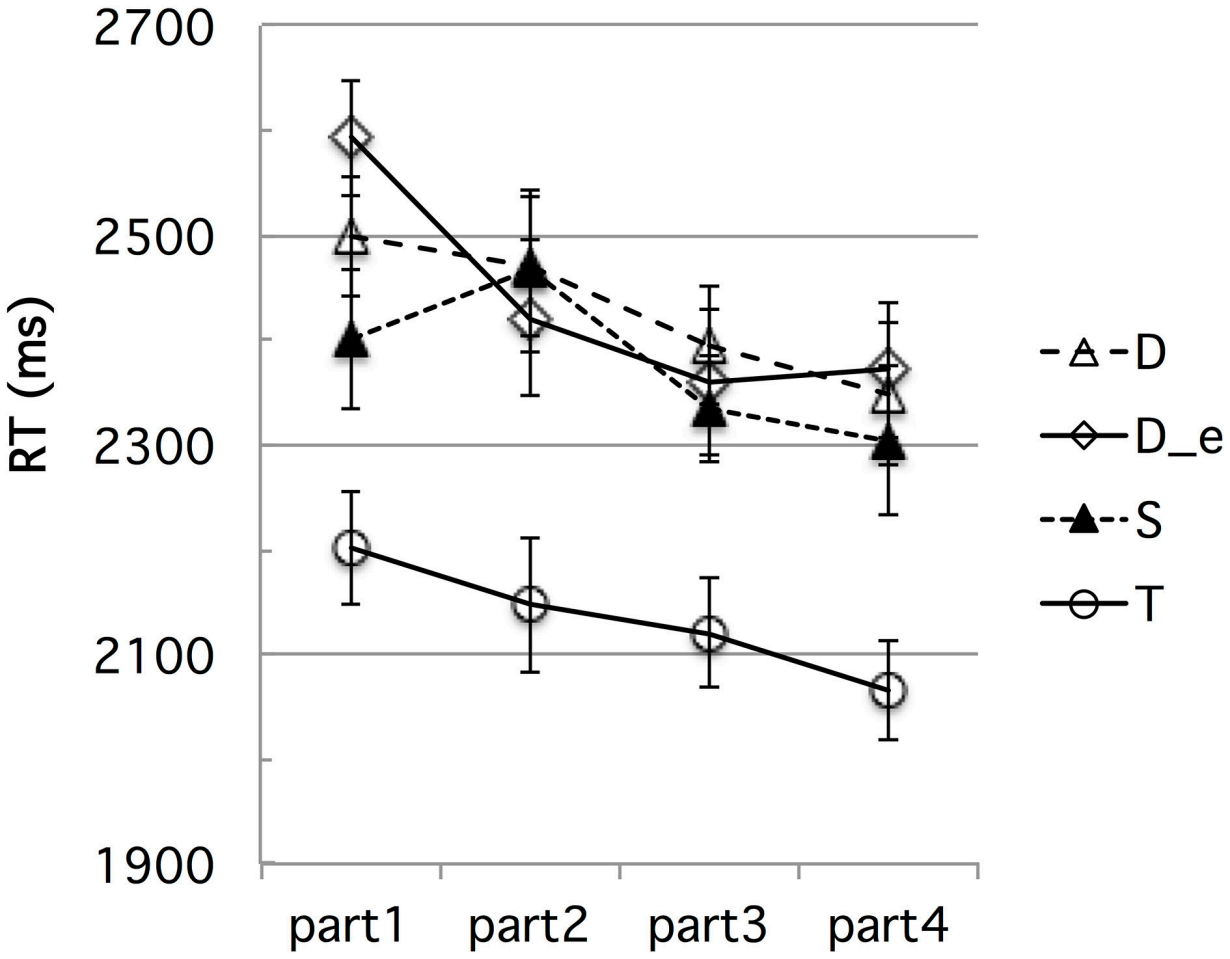
**Experiment 2: French participants' RT data for the four types of contrasts (D, D_e, S, and T for the *bi–bbi raw*, *bi–bbi equalized, sir–ssir*, and *fit–fitt* contrast types, respectively), in four successive parts of the experimental session**. The error bars represent standard errors.

We ran the same time-course analyses as in Experiment 1 to check for possible learning effects during the experimental session. Figures 7, 8 show the d-prime and RT data, respectively, in the four successive equal parts of Experiment 2.

We ran by-subject analyses of variance on these data, with the same within-subject Contrast type and session Part factors as for Experiment 1(with the addition of an “intensity-equalized voiced stop” level for the Contrast factor). For the d-prime data, the Part factor was not significant overall *F*_(3, 45)_ = 2.14, *p* = 0.11 [It approached significance for the *sir-ssir* contrast, *F*_(3, 45)_ = 2.63, *p* = 0.062]. However, Part and Contrast did not interact significantly, *F* < 1. For the RT data, the Part factor was significant *F*_(3, 45)_ = 7.63, *p* < 0.0005, showing indeed a learning effect. The Contrast x Part interaction was significant, *F*_(9, 135)_ = 2.17, *p* < 0.05, reflecting spurious differences in “learning” trajectories, especially from part 1 to part 2, as can be seen in Figure 8. Yet, importantly, RTs were much shorter for the *fit-fitt* than the other types of contrasts throughout the four session parts by an average 210 ms, *p*s < 0.00001. Thus, as in Experiment 1, we found no evidence that differential learning effects can account for the robust advantage of the *fit–fitt* contrast type over the others.

## General discussion

In this study, we first found, unsurprisingly, that French listeners generally perform much less well than Tashlhiyt listeners in discriminating Tashlhiyt geminate-singleton consonant contrasts. Tashlhiyt listeners consistently performed near ceiling level on their native contrasts. But the advantage of Tashlhiyt over French listeners varied widely across the three types of contrasts we examined. In particular, the advantage of Tashlhiyt over French listeners was smallest for word-final voiceless stop contrasts (*fit-fitt* and *hat-hatt*). In the following, we try to explain these differences.

First, it seems that French listeners could hardly use voicing murmur duration as a cue to distinguish *bi* and *bbi*. This is in line with the early cross-language work on the perception of voice onset time (VOT) continua (Abramson and Lisker, 1970, 1973). VOT continua from negative to long-lag VOTs indeed are perceived as exemplifying different categories with different categorical boundaries depending on listener's native language. For example, native speakers of Spanish segment the continuum into two VOT categories: prevoiced vs. voiceless, in agreement with their production of the Spanish contrast (Abramson and Lisker, 1973). French listeners perform very similarly (Serniclaes, 1987) presumably because French uses the same phonetic settings as Spanish to distinguish the stops of its two phonemic voicing categories. On these grounds, French listeners should not be able to discriminate well between prevoiced stops that differ in prevoicing duration.

French listeners also encountered substantial difficulty with differences in word-initial fricative duration, in spite of the notably greater audibility of friction noise than voicing murmur (see Section Stimuli and Design in Experiment 1). Common to these two types of contrasts, which French listeners had difficulty discriminating, is that they both occurred in word-initial position.

French listeners had comparatively less difficulty discriminating the word-final duration difference in the *fit*-*fitt* and *hat-hatt* contrasts, even though this duration was filled with silence, that is, even though the critical acoustic object with respect to duration was *not* audible. Why then was that condition the easiest for French listeners? Contrary to our initial prediction that singleton-geminate discrimination would be easier when carried by higher intensity portions of acoustic signal, the sole intrinsic properties of the variable-duration acoustic object at stake—silence vs. voiced murmur vs. audible friction—do not explain the observed pattern of performance. If they did, *sir-ssir* would be easier than *fit-fitt* since frication is indeed of greater intensity than silence.

Therefore, the structural difference between the word-final and word-initial contrasts seems more apt to explain the French participants' performance. One possible account may be proposed to explain the advantage of the word-final over the word-initial contrasts in terms of a differential use of the *preceding speech rate* context: The former contrasts could benefit more than the latter from the speech timing reference provided by the immediately preceding speech context. The literature on preceding speech rate context given by precursor utterances suggests robust adaptation effects (Dupoux and Green, 1997; Pallier et al., 1998) and, more recently, spectacular effects inducing the appearance vs. disappearance of phonetically reduced function words such as *or* in “*minor or child*” (Dilley and Pitt, 2010). These latter effects seem specific to a speech mode of perception, as suggested by Pitt et al. (2016), who compared the effects of sinewave speech precursors heard as speech vs. nonspeech. Previous literature has also reported syllable onset categorization effects of the *immediate, later occurring* surrounding context, in particular, current syllable rime duration (Miller and Liberman, 1979): The classic case /ba/ vs. /wa/ categorization according to syllable duration. Yet, these findings have been revisited (Shinn et al., 1985), suggesting a modulation of the effects by the natural quality of the speech. Our experimental design *did* provide a speech rate reference for timing perception in both preceding and current target speech, as well as in following speech, since each trial consists of three A, X (target), and B utterances. We nevertheless cannot definitely dismiss a speech timing reference account of our data before conducting follow up experiments specifically examining that issue.

For the moment, assuming that speech timing reference is available in our experimental design, we propose that perception of segment duration must be guided not just by the perception of the segment's acoustic substance duration (its intrinsic duration) but also, and perhaps mainly, by the perception of the timing between the successive acoustic events that bound the segment, that is, immediately precede and follow it. On this view, the word-final voiceless stop in *fit-fitt* or *hat-hatt* is clearly left-bounded by the preceding vowel /i/ or /a/ *and* right-bounded by a quite salient stop release burst (Ridouane, 2007). Conversely, the word-initial voiced stop in *bi-bbi, diR-ddiR*, or *gar-ggar*, as well as the word-initial fricative in *fit-ffit* or *sir-ssir* are clearly right-bounded by the following vowel /i/ or /a/ but they are *not saliently* left-bounded by the preceding silence. In other words, French listeners would perceive quantity distinctions more easily by detecting intervals between salient events than by “measuring” intrinsic durations. This account is also supported, however anecdotally, by the participants' performance on *jutid-juttid* during the training phase: all the Tashlhiyt participants and 83% French participants discriminated *jutid-juttid*, a medial distinction bounded on both sides by vowels. That is, the performance on *jutid-juttid* was roughly equivalent to that on *fit-fitt* or *hat-hatt*. French listeners may thus be sensitive to the beat given by successive salient phonetic events: the successive vowels /u/ and /i/ for *jutid-juttid*, the vowel /i/ or /a/ then the stop release for *fit-fitt* or *hat-hatt*.

It is tempting to think of this beat-driven perception of quantity in terms of perceptual centers or P-centers, at least a somewhat extended notion of P-centers as psychological moments of occurrence. Indeed the concept of P-centers (Morton et al., 1976) has been applied to words or syllables. What determines the “moment of occurrence” of a syllable is still debated. Some authors have proposed purely acoustically-based determinants, for example amplitude contours (in a single or several frequency bands) on the whole syllable (Howell, 1984, 1988a,b; Pompino-Marschall, 1989; Harsin, 1997) or on its onset's shape (Scott, 1993, 1998; see Vos and Rasch, 1981; Gordon, 1987, for an extension to musical tones). Other authors have proposed phonetically- or articulatory-based accounts (Fowler, 1979; Tuller and Fowler, 1980; Cooper et al., 1986; see Patel et al., 1999, for a tentative clarification). But in our speech materials, whereas P-centers naturally apply to the medial singleton vs. geminate distinctions in sequences such as *jutid* or *juttid*, so that the beat may be thought of as given by the P-centers on two successive syllables [for *ju(t)*, then *tid*], we need to extend the concept to *phonetic events* in the case of our critical test materials. This is quite straightforward in the case of sequences such as *fit* or *hatt*: both the CV part and the salient release of /t/ (singleton or geminate) may be thought of having separate moments of occurrence. In the syllables beginning with a (pre)voicing murmur or a fricative, if we attempt to define a P-center for the onset and another for the rime, the onset P-center must be weakly defined due to the low and/or slowly rising amplitude of the onset (see Vos and Rasch, 1981, for a musical tone analog). However speculative this interpretation may be, it is consistent with the idea that clearly marked left and right bounding phonetic events are needed for a “beat-based” perception of quantity.

Beat-based perception of timing may also explain the fine sensitivity to duration differences found in some other studies with French listeners, with respect to consonant duration differences in French. For example, in Spinelli et al.'s (2003), the durational differences of the /r/s in the surface homophones *dernier oignon* and *dernier rognon* are clearly bounded by the preceding and following vowels. In the case of voice-assimilated *soute* and *soude*, both surfacing as [sut] with a released [t], the duration of the final stop closure made the difference: this closure was, again, clearly bounded by the preceding vowel and the following release (Snoeren et al., 2008), a situation which is quite reminiscent of the *fit-fitt* and *hat-hatt* pairs.

For the less clearly left-bounded phonetic events (voiced closure and frication) in Tashlhiyt word-initial position, the intensity of the critical segment seems to play a role as well. Across the two experiments, there was a trend for *sir-ssir* being easier than *bi-bbi* for French listeners (though significantly so only in Experiment 1), presumably because the frication in /s/ is higher intensity, hence more audible than the voicing murmur in /b/. We therefore propose that French listeners must also be sensitive to the acoustic substance of the critical segments and that their sensitivity to sound duration differences is somewhat modulated by intensity. Yet this modulation does not seem robust. Thus, French listeners may use two different mechanisms to discriminate nonnative quantity contrasts. The first mechanism “measures” temporal gaps between salient phonetic events. The second mechanism “measures” sustained sound durations. This dichotomy may be related to early claims in the psychophysics literature suggesting that “the neural processes for the representation and comparison of filled and empty intervals may not be identical” (Fujisaki et al., 1973, p. 52; also see Abel, 1972a,b). Interestingly, sensitivity to interval gaps depends on the perceptual salience of the bounding events and is less fine-tuned (i.e., with larger just noticeable differences) than sensitivity to, for example, steady tone duration (Abel, 1972a,b).

To summarize, then, the French performance observed in this study on nonnative Tashlhiyt consonant quantity contrasts, was well below that of Tashlhiyt listeners: 73 or 70 % (experiments 1 or 2) vs. 96% correct discrimination; 2198 or 2340 ms (experiments 1 or 2) vs. 1842 ms response times. This is typical of the difficulties encountered with nonnative contrasts that have no equivalent in the native language. The Tashlhiyt performance, on the other hand, was unsurprisingly close to ceiling level. We may surmise that French perception of quantity is based on basic psychophysical processes that are universal, that is, not attuned to the specific language of the target stimuli. Hallé et al. (2004) drew similar conclusions from the French performance on Mandarin Chinese tone discrimination and identification. Whereas, the native Chinese listeners' perception of tone continua showed categorical perception properties typical of linguistically biased speech perception, the French perception of the same continua did not, and was proposed to reflect a non language-specific sensitivity to tone contours at a psychophysical level (also see Wang et al., 2001). In the present study, the French listeners' data also may reflect a psychophysical level of perception whereas the Tashlhiyt listeners' data reflect language-specific perception attuned to the phonological system of the language. Moreover, the French data suggest that one basic psychophysical mechanism can be recruited to “measure time” in speech, regardless of native language or languages. This device is based on universal sensitivity to the “beat” given by successive salient phonetic (or acoustic) events. It is presumably tightly linked to the basic capacity that is engaged in our perception of language rhythms, allowing the discrimination of languages from different rhythmic classes by human infants and adults (Nazzi et al., 1998), as well as various mammalian animals (cotton-top tamarin monkeys: Ramus et al., 2000; rats: Toro et al., 2003). Indeed, tracking rhythm, such as defined by vowel-to-vowel or syllable-to-syllable timing, seems to be at the heart of adults' or infants' sensitivity to the differential prosodic signatures of the world's languages, as proposed by Ramus and Mehler (1999).

In the case of less clearly bounded time intervals, this “beat-driven” device is logically less efficient but also seems to be complemented by a second device, which measures the duration of the phonetic or acoustic substance. This putative second device apparently requires more experience to become attuned to the phonological specificity of quantity contrasts for consonants (and, perhaps, vowels as well). Indeed the French performance is clearly lower on the contrasts that seem to mainly engage sensitivity to sustained sound duration.

As a final remark, the dichotomy we have delineated between “beat-based” and “duration-based” timing is for the time being rather speculative and should not be viewed in a strictly categorical manner. Indeed, whereas the silent occlusion in *fit-fitt* contrasts is clearly bounded by salient phonetic events, and thus fits perfectly within the definition of an event-bounded temporal gap, the voiced murmur or frication portion at the beginning of the *bi-bbi* or *sir-ssir* contrasts, respectively, are not unquestionable illustrations for sustained sounds whose durations can only be perceived from the intrinsic duration of their sound substance. Indeed, as we just argued, the beat-driven device is certainly less efficient for these contrasts but may nevertheless still be at work. Although future research is needed to substantiate our proposal, we believe that the two kinds of contrasts we examined are representative of the two ideal situations that need to be compared.

## Author contributions

All authors listed, have made substantial, direct and intellectual contribution to the work, and approved it for publication.

### Conflict of interest statement

The authors declare that the research was conducted in the absence of any commercial or financial relationships that could be construed as a potential conflict of interest.

## Footnotes

^1^Tashlhiyt has apical ([r] or [ɾ]) and uvular ([ʁ]) rhotic consonants. For sake of simplicity, we note them *r* and *R*.

^2^The models with and without this factor were not significantly different, c^2^ = 0.012, *p* = 0.89 for French, c^2^ = 1.41, *p* = 0.23 for Tashlhiyt participants.

^3^One reviewer rightly noted that the French group was older and less homogeneous in age than the Tashlhiyt group. However, we found no correlation between age and performance in the French group [% correct: *r*_(10)_ = 0.054, *p* = 0.87; RTs: *r*_(10)_ = −0.094, *p* = 0.77; d′: *r*_(10)_ = 0.251, *p* = 0.43].

^4^We also tentatively split the data into eight parts (the most we can reasonably do given that the experimental session only has 16 blocks with only one trial per contrast in each block). The time-point × performance correlations were weak and well below significance for all three relevant types of contrast (*bi-bbi*: *p* = 0.20, *sir-ssir*: *p* = 0.59, *fit-fitt*: *p* = 0.27) as well as for the filler contrasts (*p* = 0.96).
