# Corrigendum: Differential Difficulties in Perception of Tashlhiyt Berber Consonant Quantity Contrasts by Native Tashlhiyt Listeners vs. Berber-Naïve French Listeners

**DOI:** 10.3389/fpsyg.2016.00479

**Published:** 2016-03-30

**Authors:** Pierre A. Hallé, Rachid Ridouane, Catherine T. Best

**Affiliations:** ^1^Laboratoire Phonétique et Phonologie, Centre National de la Recherche ScientifiqueParis, France; ^2^Laboratoire Mémoire et Cognition, Institut National de la Santé et de la Recherche MédicaleParis, France; ^3^Haskins LaboratoriesNew Haven, CT, USA; ^4^MARCS Institute and School of Humanities and Communication Arts, University of Western SydneySydney, NSW, Australia

**Keywords:** non-native speech perception, Tashlhiyt Berber, French, geminate obstruents, timing perception

One sentence went wrong in the last paragraph of the Introduction section: “The acoustic substance …varied from silence (word-final voiceless stops: e.g., *fit-fitt*), to low-intensity voicing murmur (word-initial voiced stops: e.g., *bi-bbi*), with strident frication (word-initial fricatives: e.g., *sir-ssir*) in between.” The sentence should be: “The acoustic substance …varied from silence (word-final voiceless stops: e.g., *fit-fitt*), to strident frication (word-initial fricatives: e.g., *sir-ssir*), with low-intensity voicing murmur (word-initial voiced stops: e.g., *bi-bbi*), in between.” Indeed, our initial hypothesis is that French listeners' performance on singleton-geminate contrasts follows the critical segments' acoustic intensity. Thus, under this hypothesis, the prediction that “French listeners should encounter the greatest difficulty with voiceless stops (silence) and the least difficulty with voiceless fricatives (strident frication)” only makes sense if the ordering of the three types of segments is explained as corrected. This correction of course does not affect the scientific validity of the results.

## Author contributions

All authors listed, have contributed to this corrigendum and approved it for publication.

### Conflict of interest statement

The authors declare that the research was conducted in the absence of any commercial or financial relationships that could be construed as a potential conflict of interest.

